# Effects of small sided football and date seed (Phoenix dactylifera) powder supplementation on liver enzymes in inactive college subjects: an interventional study

**DOI:** 10.1080/15502783.2025.2532686

**Published:** 2025-07-12

**Authors:** Mohammad Mehdi Khaleghi, Abdossaleh Zar, Hamid Reza Sadeghipour

**Affiliations:** a Persian Gulf University, Department of Sport Science, School of Literature and Humanities, Bushehr, Iran; b Persian Gulf University, Persian Gulf Sports, Nutrition and Wellness Research and Technology Group, School of Literature and Humanities, Bushehr, Iran

**Keywords:** Football, date palm seed, AST, ALT, ALP, functional food

## Abstract

**Purpose:**

In recent years, the rising prevalence of metabolic disorders and liver dysfunction due to sedentary lifestyles and poor nutrition has become a growing health concern. This study investigates the effects of small sided football (SSF) and date seed powder (DSP) supplementation on liver enzymes, including aspartate aminotransferase (AST), alanine aminotransferase (ALT), alkaline phosphatase (ALP), and the ALT/AST ratio in inactive college subjects.

**Methods:**

Fifteen inactive male dormitory residents aged 21.53 ± 1.88 years (height: 174.46 ± 4.88 cm, weight: 67.67 ± 11.05 kg), who exclusively consumed university cafeteria meals, participated in two football matches. Between these matches, they consumed 0.25 g/kg of body weight of DSP thrice weekly for four weeks. Blood samples and anthropometric indices were collected before and after each game.

**Results:**

The results indicated that SSF alone led to a reduction in ALT, ALP, and the ALT/AST ratio, while increasing AST levels (*p* > 0.05). Furthermore, it was observed that supplementation with DSP resulted in an increase in ALT, AST (*p* = 0.019), and ALP, along with a decrease in the ALT/AST ratio. Additionally, the findings revealed that SSF following one month of DSP supplementation led to an increase in ALT, AST (*p* = 0.002), and ALP (*p* = 0.035), while decreasing the ALT/AST ratio.

**Conclusions:**

Four weeks of supplementation with DSP and SSF can improve liver enzyme levels, such as ALT, AST, and ALP, which indicate physiological adaptation to exercise and the potential impact of supplements on liver metabolism.

## 1. Introduction

Modern lifestyles pose a significant risk of increasing sedentary behavior, which activates pathophysiological pathways and elevates the risk of chronic diseases and premature mortality. Since the 1970s, global health organizations have sought to promote physical activity [1]; however, data from the Centers for Disease Control and Prevention (CDC) indicate that only half of adults engage in sufficient physical activity, with participation rates declining with age [2]. Medical studies highlight numerous benefits of physical activity, including reduced mortality, improved quality of life, enhanced bone and brain health, reduced anxiety, prevention of metabolic disorders, and improved liver function [1,3–5]. Nevertheless, the importance of physical activity remains inadequately understood within society [1].

Football is widely recognized as an effective means of improving cardiovascular, metabolic, and musculoskeletal fitness [6], which has led to the emergence of the concept of “Football as Medicine” in the scientific literature [7]. However, when played at high intensity, football may negatively impact various organs, including the liver, by inducing oxidative stress and lipid peroxidation [8,9]. Proper nutrition and dietary supplementation, when following scientific protocols, can help prevent nutritional deficiencies, provide energy, and enhance athletic performance. Consequently, the development of evidence-based nutritional strategies to improve health and athletic performance, particularly in football, has gained significant attention [10].

Date seed is considered a dietary supplement and has gained attention as a functional food due to its bioactive compounds. Depending on the variety and quality of the date fruit, the seed constitutes approximately 6–15% of its total weight and contains fiber, lipids, proteins, antioxidants, flavonoids, vitamins, and minerals [11]. Additionally, its polyphenols, such as catechin and procyanidin, exhibit antioxidant, antibacterial, and antiviral properties [12,13]. Given these attributes, date seed supplementation may serve as an effective strategy for improving overall health [14]. Studies have demonstrated that date seed supplementation in physically active individuals and athletes engaging in high-intensity interval training (HIIT) reduces oxidative stress and enhances performance [15]. Moreover, the combined consumption of date seed powder (DSP) and HIIT has been shown to mitigate inflammation and muscle damage [16]. However, some studies have reported varying effects of different training protocols on liver function, highlighting the need for further investigation [17].

Recent studies have increasingly highlighted the potential of plant-based compounds to modulate metabolic health markers, including liver enzymes. For instance, Bahari et al. demonstrated that pomegranate supplementation significantly improves lipid profiles, supporting the role of natural polyphenols in biochemical regulation [18]. Similarly, nut-derived bioactives have been shown to reduce inflammation and metabolic dysregulation in chronic diseases, suggesting that date seeds, which are rich in fiber and antioxidants, may exert similar functional effects [19]. Additional evidence from randomized controlled trials (RCT) indicates that natural antioxidants such as melatonin, propolis, and French maritime pine bark extract can positively influence oxidative stress, inflammation, and hepatic biomarkers [20,21]. These findings collectively support the hypothesis that date seed supplementation may modulate liver enzyme responses through antioxidant and anti-inflammatory mechanisms. Moreover, the cardiometabolic benefits of fruit-derived compounds, as highlighted in Bahari et al.‘s 2024 meta-analysis on blood pressure, further underscore the health-promoting potential of phytochemicals [22]. In this context, the current study’s combination of DSP supplementation with small-sided football – a known stimulus for metabolic adaptation – offers a novel approach to improving liver health in inactive individuals.

In recent decades, the prevalence of metabolic disorders, obesity, and liver dysfunction has risen significantly, largely due to sedentary lifestyles and unhealthy dietary patterns – an issue particularly pronounced among inactive college students engaged in predominantly sedentary academic routines. Within context, football, as a dynamic, group-based, and time-efficient physical activity, holds promise for enhancing overall health and athletic performance. Simultaneously, DSP, a functional plant-based supplement rich in antioxidant and anti-inflammatory compounds, may beneficially modulate glycemic control, lipid metabolism, liver function, and exercise-induced muscle damage. While prior studies investigating the effects of physical activity and dietary supplements on health, no research has have independently examined the health effects of either physical activity or dietary supplementation, the novelty of the present study lies in its integrative approach, assessing for the first time the synergistic impact of DSP supplementation and small-sided football (SSF) on liver enzyme responses – specifically aspartate aminotransferase (AST), alanine aminotransferase (ALT), and alkaline phosphatase (ALP) – in an understudied and at-risk population. This dual-intervention strategy not only offers a practical and accessible model for promoting hepatic health but also provides new insights into the interplay between antioxidant-rich supplementation and exercise-induced physiological adaptations.

## 2. Materials and methods

### 2.1. Ethical approval

This study was conducted at the Persian Gulf University of Bushehr, adhering to the ethical principles outlined in the Declaration of Helsinki. Before participation, all volunteers were provided with a comprehensive explanation of the study protocol and signed an informed consent form. The study protocol received approval from the Ethics Committee of Bushehr University of Medical Sciences (IR.BPUMS.REC.1403.267).

### 2.2. Participants

The sample size of this interventional study was calculated before the study using G*Power version 3.1, based on a Cohen’s f effect size of 1.05, an alpha level of 0.05, and a statistical power of 0.95. After accounting for a 10% anticipated dropout rate, the required sample size was determined to be 15 participants. This calculation ensured sufficient power to detect significant effects in the context of our design.Between November and December 2024, fifteen inactive male undergraduate students with prior experience in playing football voluntarily participated in this study. The study protocol adhered to the Consolidated Standards of Reporting Trials (CONSORT) checklist. The inclusion criteria were as follows: being in good health, having familiarity and experience with football, not engaging in regular physical activity, residing in dormitories, consuming three cooked meals provided by the university cafeteria, and willingness to participate in the study. Although participants may have come from different socio-economic backgrounds, their shared residential setting and uniform dietary conditions helped ensure a relatively homogeneous socio-economic status and nutritional intake. These controlled conditions minimized potential confounding effects related to socio-economic disparities and dietary variability. Consequently, a detailed food intake chart was not generated, as all participants followed the same dietary regimen with no individual deviations. The exclusion criteria included a history of morphological or cardiovascular disorders, significant orthopedic issues, musculoskeletal injuries, consumption of ergogenic dietary supplements (e.g. creatine, β-hydroxy-β-methylbutyrate, etc.), various antioxidants, anabolic/catabolic hormones (e.g. androstenedione, dehydroepiandrosterone, etc.), antacids, antibiotics, antidiarrheal and anti-inflammatory medications, smoking, and adherence to a special or restricted diet within six months before the study.

### 2.3. Experimental design

After the recruitment process and obtaining written informed consent, all participants (*n* = 15) were enrolled in a single-group, repeated-measures experimental design. The study included two exercise trials (SSF matches), conducted before and after a one-month dietary supplementation phase. To minimize confounding variables, participants were instructed to abstain from any vigorous physical activity for at least 48 hours prior to each exercise session. On the first experimental day, following an overnight fast (beginning at midnight), baseline blood samples were collected at 8:00 AM. Anthropometric measurements, including height and weight, were taken using a calibrated scale and stadiometer (Seca Co., Germany). Participants were then provided with a standardized breakfast (sandwich with bread, cheese, tomato, and cucumber), followed by participation in an SSF match. Immediately after the match, post-exercise blood samples were collected.

**Figure 1.**
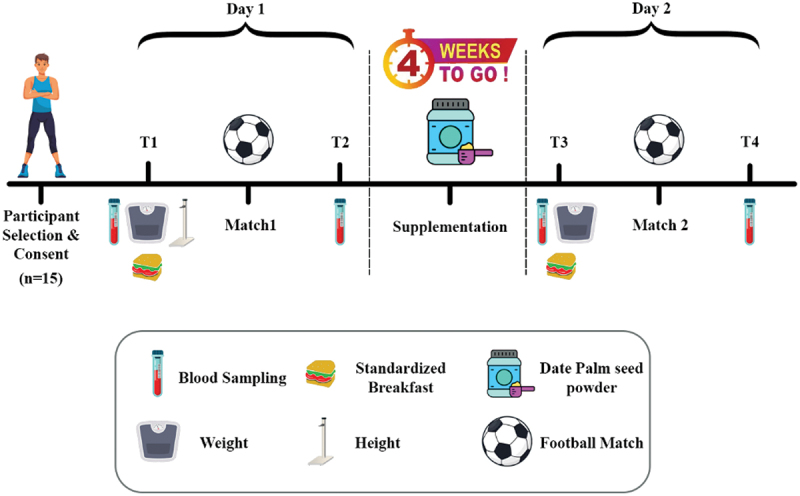
Experimental design.

Subsequently, after a 24-hour rest period, participants underwent a one-month supplementation protocol with DSP, administered three times per week according to a standardized dosage. After completing the supplementation period and ensuring a 24-hour washout period following the final DSP dose, the second experimental session was conducted. Similar to the first phase, blood samples were collected both before and immediately after an SSF match, and body weight was remeasured. All collected blood samples were subsequently transported under controlled conditions to the laboratory for biochemical analysis (Figure 1).

### 2.4. Intervention

The participants consumed DSP supplementation (Flavinea Co.) at a dosage of 0.25 g/kg of body weight, dissolved in 250 mL of water, three times per week on nonconsecutive days for one month [23]. To ensure adherence to the supplementation protocol, participants visited the researcher to receive their supplement doses for each session, and consumption was supervised directly by the principal investigator. Additionally, the principal investigator maintained daily contact with the participants to address any potential concerns regarding supplementation.

### 2.5. Exercise protocol

The training sessions were conducted as small-sided games (2 vs. 2, 3 vs. 3, and 4 vs. 4) using 1.2 × 5 m goals, with rotating goalkeepers. These games are recognized as an intense and dynamic training method for untrained individuals, incorporating rapid forward, backward, and lateral runs, as well as high-intensity movements such as dribbling, shooting, and turning [24,25]. Each football session began with a standardized warm-up, consisting of 1.5 minutes of low-intensity intermittent running, including the first six 20-meter shuttles from the Yo-Yo Intermittent Endurance Test Level 1 [26]. Each 40-meter run was followed by 5 seconds of active recovery, during which participants walked twice over a 2.5-meter distance. The sessions were supervised by the researcher and university staff, while the players acted as referees. The one-hour football session was divided into four 13.5-minute bouts, each followed by a 1.5-minute recovery period [27].

### 2.6. Body composition

Anthropometric parameters, including height, weight, and body mass index (BMI), were assessed at both the beginning and the end of the study. Weight and height were measured using a validated scale and stadiometer, with participants wearing light clothing and no shoes, and recorded to the nearest 0.5 kg and 0.1 cm. BMI was calculated by dividing weight (in kilograms) by the square of height (in meters). According to the World Health Organization, BMI is considered a standard metric for determining nutritional status [28].

### 2.7. Blood samples

At each session, blood samples were collected from participants both before and immediately after completing the exercise protocol. Specifically, 5 cc of venous blood was drawn from the right arm and transferred into tubes with or without ethylenediaminetetraacetic acid. The blood samples were then centrifuged and subsequently transported to the laboratory for analysis of the targeted liver enzymes, including ALT, AST, and ALP, by an **autoanalyzer** using kits (D**e**lta Darman Part Co.) [29].

### 2.8. Chemical composition and antioxidant properties of date seed powder

Commercially prepared DSP samples were obtained from Flavinea Co. The chemical composition of DSP was analyzed following the standard protocols outlined by the Association of Official Analytical Chemists [30]. To quantify the total phenolic content, the Folin – Ciocalteu colorimetric method was employed [31], while flavonoid concentration was determined using the aluminum chloride colorimetric assay [32]. Table 1 presents the mean composition and antioxidant characteristics of DSP (per 100 g), with all measurements conducted in triplicate [15] (Table 1).

**Table 1.** Chemical profile, total phenolic acid, and flavonoid content of date seeds and placebo (per 100 g and per supplement package).

Name		Date seeds (n = 3)	
		Mean	SD
Energy (kcal)		270.79	21.65
Carbohydrate (g)		13.12	2.56
Protein (g)		6.10	0.82
Total lipids (g)		5.70	1.03
Fiber total (g)		66.76	10.43
Soluble dietary fiber (g)		57.12	11.48
Insoluble dietary fiber (g)		9.64	2.06
Ash (g)		1.30	0.11
Moisture (%)		7.02	1.42
Antioxidants	Total phenolic acid (mg GAE/g)	3456.86	522.71
	Flavonoid content (mg QE/g dry weight)	1624.54	352.12

Kcal: Kilocalorie; g: Gram; mg: Milligram; GAE: Gallic acid equivalent; QE: Quercetin equivalent.

### 2.9. Statistical analysis

The Shapiro – Wilk test was employed to assess the normality of the data distribution. The mean and standard deviation were used to statistically describe the data. Mean comparisons were conducted using analysis of variance (ANOVA), followed by Scheffé’s post hoc procedure to identify pairwise differences. Additionally, Pearson’s correlation analysis was employed to assess the relationships among the various measured variables. Data analysis was conducted using SPSS statistical software (version 24; SPSS Inc., Chicago, IL, USA), **with** a significant level **set at** *p* ≤ 0.05.

## 3. Results

### 3.1. Participants characteristics

The 15 participants in this study had a mean age of 21.53 ± 1.88 years (range: 19–25 years) and a mean height of 174.46 ± 4.88 cm. Analysis of weight and BMI changes before and after the intervention indicated a significant increase in both parameters (weight: 67.67 ± 11.05 kg vs. 69.31 ± 10.98 kg, *p* < 0.001; BMI: 22.21 ± 3.36 kg/m^2^ vs. 22.75 ± 3.33 kg/m^2^, *p* < 0.001) (Table 2).

**Table 2.** Participants characteristics.

Factors	Baseline (T1)	Pre-test 2 (T3)	P
**Age** (years)	21.53 ± 1.88	−	−
**Height** (cm)	174.46 ± 4.88	−	−
**Weight** (kg)	67.67 ± 11.05	69.31 ± 10.98	<.001
**BMI** (kg/m^2^)	22.21 ± 3.36	22.75 ± 3.33	<.001

Data are shown as mean ± standard deviation. Cm: Centimeter; kg: Kilogram; Baseline (T1): Baseline score in the first pretest before the start of the first small sided football (SSF); Pretest 2 (T3): Second pretest before the start of the second SSF after a supplementation period.

### 3.2. Liver enzymes changes

**Figure 2.**
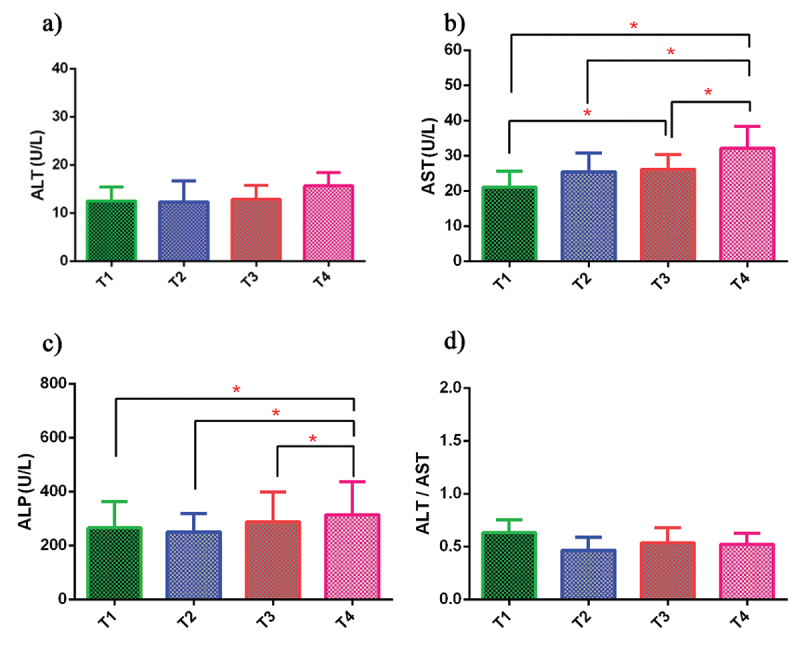
Comparison of ALT (a), AST (b), and ALP (c) enzyme levels, and the ALT/AST ratio (d), between groups under different conditions and time points of the research protocol. Baseline (T1): baseline score in the first pre-test before the start of the first small sided football (SSF); post-test 1 (T2): first post-test after the first SSF; pre-test 2 (T3): second pre-test before the start of the second SSF after a supplementation period; post-test 2 (T4): second post-test after the second SSF after a supplementation period; *: significant difference at the *p* < 0.05 level; data are presented as the mean ± standard error of the mean.

Liver enzyme levels, including ALT, AST, ALP, and the ALT/AST ratio, measured at different time points, are presented in Table 3. AST levels increased after the first match, with further statistically significant elevations observed prior to the second match (p = 0.019) and immediately afterward (p = 0.002). The ALP concentration decreased following the first match but showed an increase after one month of supplementation both immediately prior to the second match and immediately after the second match (T4), with the increase at T4 reaching statistical significance (p = 0.035). No statistically significant changes were observed in ALT concentrations or in the ALT/AST ratio across the measured time points (Figure 2).

**Table 3.** Liver enzymes changes at Baseline, post test 1, pre test 2, and post test 2.

Factors	Baseline (T1)	Post-test 1 (T2)	Pre-test 2 (T3)	Post-test 2 (T4)
**ALT** (U/L)	12.50 ± 2.95	12.30 ± 4.40	12.90 ± 2.88	15.70 ± 2.75
**AST** (U/L)	21.09 ± 4.59	25.45 ± 5.28	26.18 ± 4.21^a^	32.18 ± 6.19^b c d^
**ALP** (U/L)	265.40 ± 97.06	250.53 ± 67.97	288.73 ± 110.64	314.80 ± 122.16^b c d^
**ALT/AST**	0.63 ± 0.12	0.47 ± 0.12	0.54 ± 0.14	0.52 ± 0.10

Data are shown as mean ± standard deviation.

**ALT**: Alanine aminotransferase; **AST**: Aspartate aminotransferase; **ALP**: Alkaline Phosphatase; **U/L**: Units per liter; **Baseline (T1)**: Baseline score in the first pre-test before the start of the first small sided football (SSF); **Post-test 1 (T2)**: First post-test after the first SSF; **Pre-test 2 (T3)**: Second pre-test before the start of the second SSF after a supplementation period; **Post-test 2 (T4)**: Second post-test after the second SSF after a supplementation period.

ANOVA with Scheffe´ Test: T_1_ vs. T_3_: ^a^*p* < 0.05, T_1_ vs. T_4_: ^b^*p* < 0.05, T_2_ vs. T_4_: ^c^*p* < 0.05, T_3_ vs. T_4_: ^d^*p* < 0.05.

## 4. Discussion

The present study investigated the liver enzymes Response to one-month Date Seed (Phoenix dactylifera) Powder Supplementation and a SSF in Inactive College Students. Our findings demonstrated that supplementation with DSP for four weeks increased ALT, AST, and ALP enzymes and attenuated the ALT/AST ratio in Inactive male collegian**s**.

Since physical activity increases oxygen consumption, it is hypothesized that this condition may enhance the production of free radicals and contribute to oxidative stress [33]. Furthermore, as previous studies have classified small-sided games (SSGs) as a form of HIIT [34], it can be inferred that participation in small-sided football games may induce oxidative stress and lipid peroxidation through mechanisms such as the activation of NADPH oxidase, xanthine oxidase, and phospholipase A2, as well as cytochrome c release from mitochondria and catecholamine oxidation [8,9].

Additionally, research has demonstrated that physical activity can affect the levels of hepatic enzymes such as ALT and AST. In addition to their metabolic roles, these enzymes serve as biomarkers of tissue injury and cellular necrosis [35], and are typically released into the circulation in response to exercise-induced muscle damage, depending on the intensity and duration of physical exertion [36]. However, under certain conditions – such as structured exercise protocols or SSGs, enzyme levels may exhibit variations or remain within stable ranges [37–39].

Overall, ALT and AST, which are released from the liver into muscles, play a crucial role in protein metabolism. Fluctuations in their serum concentrations may reflect the extent of muscular stress induced by physical activity [40]. On the other hand, several adaptive responses – such as redox signaling cascades, regulation of endogenous antioxidant enzymes, skeletal muscle glucose uptake, and mitochondrial biogenesis – have been shown to mitigate oxidative stress following such training protocols [41]. Nevertheless, to further enhance the body’s antioxidant capacity, supplementation with a flavonoid- and antioxidant-rich compound such as DSP may be beneficial in protecting against oxidative damage.

The observed increase in hepatic enzymes – ALT, AST, and ALP – along with the reduction in the ALT/AST ratio following DSP supplementation, supports the hypothesis that phenolic compounds and carbohydrate constituents with absorptive properties in date seed extract, as well as its modulatory effects on gene expression, may contribute to its antioxidant and anti-inflammatory activity. Specifically, date seed extract has been reported to upregulate the expression of Interferon gamma and Interleukin-2 (key macrophage activators), as well as antioxidant enzymes such as Glutathione peroxidase, and Superoxide dismutase (SOD), while downregulating the expression of the pro-inflammatory gene iNOS [42]. These molecular effects may underlie the observed biochemical changes. In this regard, several studies have demonstrated that the consumption of various plant-based compounds – such as pomegranate and nuts – may positively influence physiological and metabolic health. Owing to their rich content of polyphenols and other bioactive constituents, these natural products have been shown to improve inflammatory status and metabolic dysregulation associated with chronic diseases and diverse physiological conditions [18,19].

Furthermore, the improvement in hepatic enzyme levels following date seed supplementation may be attributed to its antioxidant potential. Specifically, the potent antioxidant compounds present in date seeds, including flavonoids and active phenolic constituents, may stabilize hepatic cell membranes, thereby preventing the leakage of hepatic enzymes into the bloodstream [43,44]. Additionally, these compounds may alleviate oxidative stress in the liver by scavenging reactive oxygen species (ROS), thus mitigating oxidative stress-related hepatic injury and diabetes-associated liver dysfunction [45,46]. In this context, several RCTs have provided indirect evidence that natural antioxidants – such as melatonin, propolis, and French maritime pine bark extract – can exert beneficial effects on oxidative stress, inflammation, and hepatic biomarkers [20,21].

Moreover, the bioactive compounds found in date seeds have been shown to inhibit various inflammatory mediators, such as TNF-α and NF-κB, thereby exerting hepatoprotective effects through the preventing of inflammation [47]. Specifically, date seed extract has been reported to significantly reduce levels of multiple pro-inflammatory cytokines, including TNF-α [48], subsequently leading to the suppression of NF-κB signaling [49]. Additionally, rutin, quercetin, p-coumaric acid, and caffeic acid have been identified as the predominant polyphenols among the phenolic compounds extracted from date seeds, with a significant correlation observed between these polyphenols and the anti-inflammatory effects of date seed extract [47].

Furthermore, multiple studies have demonstrated that pretreatment with p-coumaric, caffeic, chlorogenic, gallic, syringic, and vanillic acids, as well as rutin, luteolin, and quercetin – compounds naturally present in date seeds – can prevent pathological elevations in AST and ALT levels. These protective effects are mediated through the enhancement of hepatic antioxidant capacity, as indicated by increased activity of key antioxidant enzymes such as SOD, GSH, and CAT [50–58].

Another study demonstrated that the ability of date seeds to reverse elevated serum liver enzyme levels and preserve the histopathological characteristics of the liver may be attributed to their capacity to lower elevated serum glucose levels. This glycemic reduction, in turn, helps mitigate oxidative stress and prevent the metabolic alterations associated with hyperglycemia [46]. Several mechanisms contribute to hyperglycemia-induced tissue damage, including increased glucose flux through the polyol pathway, excessive intracellular production of advanced glycation end-products (AGEs), upregulation of AGE receptors, activation of **specific isoforms of protein kinase C**, and induction of the hexosamine **biosynthetic** pathway. Numerous studies suggest that these mechanisms are driven by a common initial event – overproduction of mitochondrial ROS [59,60]. Furthermore, date seeds have been shown to downregulate hepatic expression of the pro-apoptotic protein caspase-3, which may represent another key mechanism underlying their hepatoprotective effects [61].

### 4.1. Strengths and limitations

This study presents several novel contributions. First, its interventional design combined with precise biochemical assessments enhances both the reliability and validity of the findings. Additionally, the homogeneity of participants with respect to dietary patterns and physical activity levels – alongside strict monitoring of supplement intake, compliance with dietary guidelines, and avoidance of additional physical activity – helped to minimize confounding variables and strengthen the study’s credibility. Moreover, the innovative aspect of investigating DSP as a natural, cost-effective supplement in a sports intervention underscores its potential benefits. Utilizing this agricultural byproduct could optimize resource use, reduce waste management costs, and improve nutritional strategies in sports.

However, this study has some limitations. First, the absence of a body composition analyzer prevented precise assessment of changes in body composition before and after the intervention. Future studies should employ advanced measurement tools to assess these parameters more accurately. Second, the method of DSP consumption posed a challenge, suggesting the need for simpler and more acceptable intake methods. Furthermore, exploring different dosages and supplementation durations across diverse populations could help establish an optimal protocol for DSP usage.

## 5. Conclusion

The findings of this study indicate that four weeks of supplementation with DSP influenced hepatic enzyme responses following an SSF. Specifically, ALT, AST, and ALP levels increased after the intervention and exercise, suggesting physiological adaptations to training and the potential impact of supplementation on liver metabolism. Furthermore, the ALT/AST ratio fluctuated throughout the study, with significant reductions observed at certain time points. These findings highlight the interactive effects of football game and DSP supplementation on liver function biomarkers.
